# Molecular and solid-state topological polaritons induced by population imbalance

**DOI:** 10.1515/nanoph-2023-0158

**Published:** 2023-06-12

**Authors:** Sindhana Pannir-Sivajothi, Nathaniel P. Stern, Joel Yuen-Zhou

**Affiliations:** Department of Chemistry and Biochemistry, University of California San Diego, La Jolla, CA 92093, USA; Department of Physics and Astronomy, Northwestern University, Evanston, IL 60208, USA

**Keywords:** exciton–polariton, strong light–matter coupling, topological polaritons

## Abstract

Strong coupling between electronic excitations in materials and photon modes results in the formation of polaritons, which display larger nonlinearities than their photonic counterparts due to their material component. We theoretically investigate how to optically control the topological properties of molecular and solid-state exciton–polariton systems by exploiting one such nonlinearity: saturation of electronic transitions. We demonstrate modification of the Berry curvature of three different materials when placed within a Fabry–Perot cavity and pumped with circularly polarized light, illustrating the broad applicability of our scheme. Importantly, while optical pumping leads to nonzero Chern invariants, unidirectional edge states do not emerge in our system as the bulk-boundary correspondence is not applicable. This work demonstrates a versatile approach to control topological properties of novel optoelectronic materials.

## 1 Introduction

Exciton–polaritons are hybrid excitations that exist in systems where photonic modes couple strongly with optical transitions in materials and their coupling strength exceeds losses [1]. Electronic strong coupling (ESC), where the optical transitions correspond to semiconductor excitons or molecular electronic transitions, has been observed in a wide variety of inorganic and organic materials. While some polariton systems, such as GaAs and CdTe quantum wells in microcavities [1, 2], often require cryogenic temperatures for operation, due to their small exciton binding energies, organic materials [3] along with others such as GaN [4], ZnO [5], perovskites [6, 7], and transition metal dichalcogenides (TMD) [8, 9] can achieve ESC at room temperature when placed in Fabry–Perot cavities. In particular, organic exciton–polaritons have received attention for their ability to modify chemical reactivity [10], demonstrate polariton condensation at room temperature [11, 12], improve photoconductivity [13], and display topological properties [14, 15].

Exciton–polariton systems are versatile platforms for topological applications as their hybrid nature provides the unique opportunity to take advantage of the nonlinearities and magnetic response of the material component while still enjoying benefits of the coherence properties of the photonic part [16–18]. In the presence of photonic lattices, they also offer the possibility of unidirectional transport of energy through edge states that are robust to disorder [19]. A few approaches are frequently used to achieve topological exciton–polariton bands. In one of the approaches, the nontrivial topology resides in the winding light–matter coupling rather than individual photon or exciton components [19, 20]. However, it is limited in application due to the requirement of large magnetic fields to break time-reversal symmetry (TRS) and low temperatures to achieve Zeeman splitting in the exciton component, which exceeds the exciton linewidth. In another approach, TRS is preserved and a quantum spin hall insulator analog is created in a polariton system [21]. This approach does not require a large magnetic field; however, there, a topological polariton system is created by coupling a topologically nontrivial photonic lattice with a topologically trivial exciton system and the interesting topology is almost entirely encoded in the photonic component of the polariton [21, 22]. Both the approaches mentioned above were experimentally realized in polariton lattices. More recently, polaritons in Fabry–Perot cavities have emerged as a viable platform for topological polaritonics. Several experiments have demonstrated measurement and control of the Berry curvature of exciton–polariton and photon bands in these systems [23–26]. Our work will focus on these Fabry–Perot cavity systems.

In this work, we theoretically propose a scheme for generating topological polaritons that combines advantages of both the approaches mentioned above. Specifically, we exploit the primary nonlinearity of organic exciton–polaritons, saturation [11], to achieve this. Here, the light–matter coupling contains the nontrivial topology instead of the individual photon or exciton components and optical pumping with circularly polarized light breaks TRS instead of a large magnetic field.

Breaking TRS in a system using the helicity of light is an idea that has been demonstrated in several other contexts; it has been used to achieve all-optical nonreciprocity [27, 28], and theoretical results suggest that it can also induce optical activity in achiral molecules [29]. Additionally, a similar idea that relies on breaking TRS using circularly polarized light has been previously proposed for polariton lattices by Bleu et al. [30].

We focus on the topological properties of polaritons formed by the coupling of Frenkel excitons hosted in organic semiconductors with photon modes in a Fabry–Perot cavity. Here, optical pumping with circularly polarized light saturates certain electronic transitions and breaks TRS in the system; this results in nonzero Chern numbers of polariton bands. Our scheme relies on the contraction of Rabi splitting due to saturation, and we find modified Berry curvature and Chern number of the bands under circularly polarized pumping. The Berry curvature of the more photonic sections of the bands computed in our work can be experimentally measured using pump-probe spectroscopy. Furthermore, the applicability of our scheme is not limited to organic polariton systems. It only requires certain key ingredients: transitions that can be selectively excited with circularly polarized light, saturation effects, and Rabi splitting contraction. To highlight this, we compute the Berry curvature of two other systems under strong coupling and optical pumping: (a) Ce:YAG and (b) monolayer MoS_2_. Our work provides a viable strategy to induce nonreciprocal behavior in standard microcavity polaritons, leading to the optical tuning of isolators and circulators [27], as well as fabrication of elliptically polarized lasers and condensates [31].

## 2 Results

### 2.1 Model

**Figure 1:**
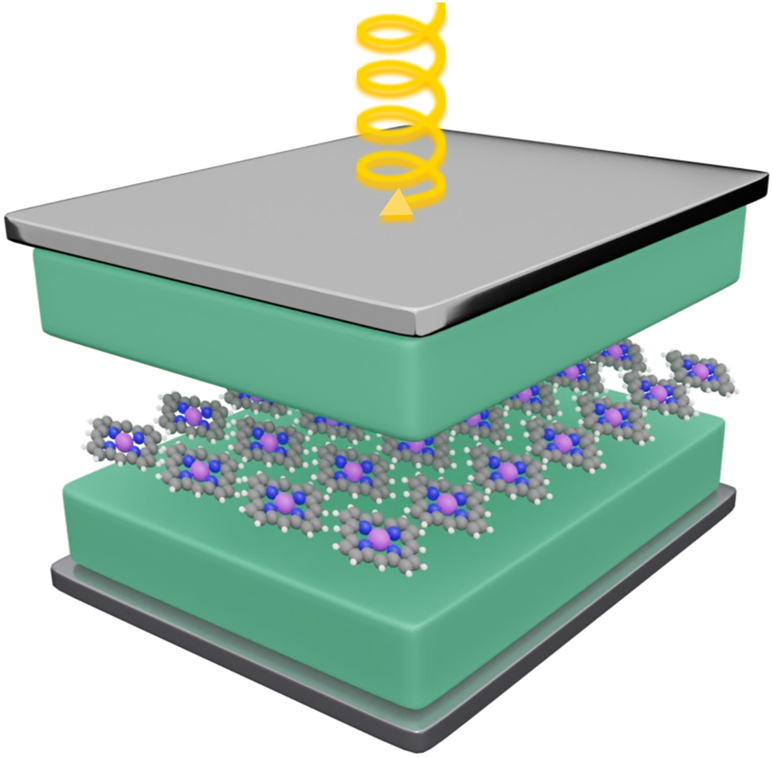
Illustration of the system under study. Porphyrin (molecules at the center) and perylene (green blocks) placed within a Fabry–Perot cavity and pumped with circularly polarized light.

In our theoretical study, we consider a Fabry–Perot cavity containing a thin film of porphyrin molecules at the center and a bulk perylene crystal filling the rest of the volume (Figure 1). The porphyrin and perylene molecules are not treated on an equal footing in our model; while the molecular transitions of porphyrin are considered explicitly in the Hamiltonian, those of the perylene crystal are not, and they can be accounted for through effective cavity modes [25]. This is a valid approximation because we focus on photon modes with frequencies close to those of electronic transitions in porphyrin ( ∼3.81 eV) [32, 33] and far off-resonant from the transitions of perylene ( ∼2.98 eV) [34]. Here, the birefringent perylene crystal plays the role of providing anisotropy and emergent optical activity to the cavity modes [25].

**Figure 2:**
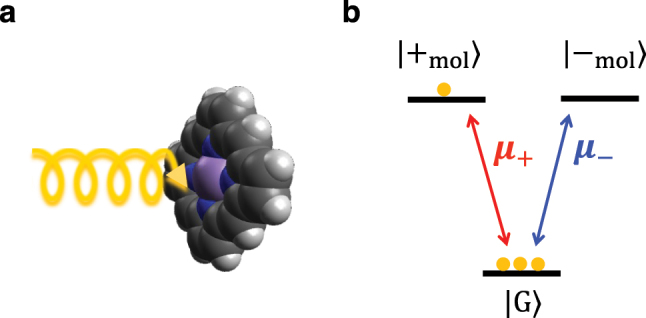
Three-level model of a metalloporphyrin molecule. (a) Illustration of circularly polarized light exciting a metalloporphyrin molecule. (b) Three-level model of porphyrin with a ground state $\left|G\right\rangle$ and two degenerate excited states $\left|{+}_{\text{mol}}\right\rangle ,\left|{-}_{\text{mol}}\right\rangle$ . The transition dipole moment for a transition from $\left|G\right\rangle$ to $\left|\pm_{\text{mol}}\right\rangle$ is $\mu_{\pm }=\mu_{0}\left(\hat{x}\pm i\hat{y}\right)/\sqrt{2}$ . The number of yellow circles at each state represents the fraction of molecules in that state. Here, the ratio of the fraction of molecules in the ground, *f*_G_, and $\left|\pm_{\text{mol}}\right\rangle$ excited states, *f*_±_, is *f*_G_ : *f*_+_ : *f*_−_ = 3 : 1 : 0. Such population ratios can be achieved through pumping with circularly polarized light.

We model each porphyrin molecule as a three-level electronic system with a ground state $\left|G\right\rangle$ and two excited states $\left|{+}_{\text{mol}}\right\rangle$ and $\left|{-}_{\text{mol}}\right\rangle$ (see Figure 2b) [35, 36]. In the absence of a magnetic field, the two excited states are degenerate and the energy difference between the ground and excited states is *ℏω*_e_ = 3.81 eV [37]. The transition dipole moments for transitions from $\left|G\right\rangle$ to $\left|{+}_{\text{mol}}\right\rangle$ and $\left|{-}_{\text{mol}}\right\rangle$ are $\mu_{+}=\mu_{0}\left(\hat{x}+i\hat{y}\right)/\sqrt{2}$ and $\mu_{-}=\mu_{0}\left(\hat{x}-i\hat{y}\right)/\sqrt{2}$ , respectively, with *μ*_0_ = 2.84D [37]. Here, $\hat{x}$ and $\hat{y}$ are unit vectors along the *x* and *y* directions. Using circular polarized light, the $\left|{+}_{\text{mol}}\right\rangle$ or $\left|{-}_{\text{mol}}\right\rangle$ states can be selectively excited.

In our model, we consider a thin film of metalloporphyrins or metallophtalocyanines arranged in a square lattice with nearest neighbor spacing *a*. The choice of lattice is irrelevant because later we will take the continuum limit *a* → 0 as we are only interested in length scales much larger than the intermolecular spacing. Additionally, we use periodic boundary conditions along the *x* and *y* directions and consider a box of size *L*_*x*_ × *L*_*y*_. Each molecule is labeled with the index **m** = (*m*_*x*_, *m*_*y*_) that specifies its location in a *N*_*x*_ × *N*_*y*_ array of molecules where *L*_*x*/*y*_ = *aN*_*x*/*y*_; here, the molecule’s position is given by $r_{m}=m_{x}a\hat{x}+m_{y}a\hat{y}$ . States of the **m**th molecule are then written as $\left|m,G\right\rangle$ , $\left|m,{+}_{\text{mol}}\right\rangle$ and $\left|m,{-}_{\text{mol}}\right\rangle$ . The creation operator ${\hat{\sigma }}_{m,\pm }^{†}=\left|m,\pm_{\text{mol}}\right\rangle \left\langle m,G\right|{⊗}_{n\neq m}I_{n}$ excites the **m**th molecule from $\left|m,G\right\rangle$ to $\left|m,\pm_{\text{mol}}\right\rangle$ . Here, $I_{n}=\left|n,G\right\rangle \left\langle n,G\right|+\left|n,{+}_{\text{mol}}\right\rangle \left\langle n,{+}_{\text{mol}}\right|+\left|n,{-}_{\text{mol}}\right\rangle \left\langle n,{-}_{\text{mol}}\right|$ is the identity operator for **n**th molecule. These molecular operators satisfy commutation relations (a generalization of the commutation relations of paulion operators [38, 39]),

(1) $$\left[{\hat{\sigma }}_{n,\pm },\;{\hat{\sigma }}_{m,\pm }^{†}\right]=\delta_{m,n}\left(1-{\hat{\sigma }}_{n,\mp }^{†}{\hat{\sigma }}_{n,\mp }-2{\hat{\sigma }}_{n,\pm }^{†}{\hat{\sigma }}_{n,\pm }\right).$$

We model the effective photon modes of a Fabry–Perot cavity filled with perylene as in Ren et al. [25]. For the photon modes of a Fabry–Perot cavity, the component of wave vector orthogonal to the mirrors *k*_*z*_ = 2*n*_*z*_ *π*/*L*_*z*_ is quantized, where *L*_*z*_ is the effective distance between the mirrors of the cavity and *n*_*z*_ is the mode index [40]. For a given *n*_*z*_, the modes are labeled by the in-plane wave vector $k=k_{x}\hat{x}+k_{y}\hat{y}$ and polarization *α*; the creation operators associated with these modes are ${\hat{a}}_{k,\alpha }^{†}$ and they satisfy bosonic commutation relations $\left[{\hat{a}}_{k,\alpha },\;{\hat{a}}_{k^{\prime },\alpha^{\prime }}^{†}\right]=\delta_{\alpha ,\alpha^{\prime }}\delta_{k,k^{\prime }}$ . As a result of in-plane translational invariance of the cavity and periodic boundary conditions along the *x* and *y* directions, *k*_*x*_ = 2*l*_*x*_ *π*/*L*_*x*_ and *k*_*y*_ = 2*l*_*y*_ *π*/*L*_*y*_ take a discrete but infinite set of values $l_{x},l_{y}\in Z$ . Throughout this work, we specify the cavity mode polarization in the circularly polarized basis *α* = ±.

The Hamiltonian of the full system is

(2) $$\hat{H}={\hat{H}}_{\text{mol}}+{\hat{H}}_{\text{cav}}+{\hat{H}}_{\text{cav}-\text{mol}},$$

where

(3) $$\begin{array}{ll} {\hat{H}}_{\text{mol}} & =\underset{m}{\sum }\left(\hbar \omega_{\text{e}}{\hat{\sigma }}_{m,+}^{†}{\hat{\sigma }}_{m,+}+\hbar \omega_{\text{e}}{\hat{\sigma }}_{m,-}^{†}{\hat{\sigma }}_{m,-}\right)\\ {\hat{H}}_{\text{cav}} & =\underset{k}{\sum }\left[\left(E_{0}+\frac{\hbar^{2}|k{|}^{2}}{2m^{*}}+\zeta |k|\cos\phi \right){\hat{a}}_{k,+}^{†}{\hat{a}}_{k,+}\right.\\ & \;+\left(E_{0}+\frac{\hbar^{2}|k{|}^{2}}{2m^{*}}-\zeta |k|\cos\phi \right){\hat{a}}_{k,-}^{†}{\hat{a}}_{k,-}\\ & \;+\left(-\beta_{0}+\beta |k{|}^{2}{\text{e}}^{-\text{i}2\phi }\right){\hat{a}}_{k,+}^{†}{\hat{a}}_{k,-}\\ & \;+\left.\left(-\beta_{0}+\beta |k{|}^{2}{\text{e}}^{\text{i}2\phi }\right){\hat{a}}_{k,-}^{†}{\hat{a}}_{k,+}\right],\\ {\hat{H}}_{\text{cav}-\text{mol}} & =\underset{m}{\sum }\underset{k,\alpha }{\sum }-{\hat{\mu }}_{m}\cdot {\hat{E}}_{k,\alpha }\left(r_{m},0\right)\\ & \approx \underset{m}{\sum }\underset{k}{\sum }\frac{{\text{e}}^{\text{i}k\cdot r_{m}}}{\sqrt{N_{x}N_{y}}}\left[\left(\mu_{+}\cdot J_{k,+}\right){\hat{\sigma }}_{m,+}^{†}{\hat{a}}_{k,+}\right.\\ & \;+\left(\mu_{-}\cdot J_{k,+}\right){\hat{\sigma }}_{m,-}^{†}{\hat{a}}_{k,+}+\left(\mu_{+}\cdot J_{k,-}\right){\hat{\sigma }}_{m,+}^{†}{\hat{a}}_{k,-}\\ & \;+\left.\left(\mu_{-}\cdot J_{k,-}\right){\hat{\sigma }}_{m,-}^{†}{\hat{a}}_{k,-}\right]+H.c. \end{array}$$

Above, ${\hat{H}}_{\text{mol}}$ describes the porphyrin molecules, ${\hat{H}}_{\text{cav}}$ the effective cavity modes (including contributions from the perylene crystal), and ${\hat{H}}_{\text{cav}-\text{mol}}$ the coupling between the porphyrin molecules and effective cavity modes. Here, *ϕ* is the angle between the in-plane wave vector and the *x*-axis, *i.e.*, cos *ϕ* = *k*_*x*_/|**k**|. Within ${\hat{H}}_{\text{cav}}$ , *β* specifies the TE-TM splitting, *β*_0_ quantifies the linear birefringence of the perylene crystal which splits the H–V modes, and *ζ* describes the emergent optical activity [25]. Additionally, *E*_0_ is the frequency of the cavity modes at |**k**| = 0 in the absence of the perylene crystal (*β*_0_ = 0 and *ζ* = 0), and m* is the effective mass of the photons in the absence of perylene (*β*_0_ = 0 and *ζ* = 0) and TE-TM splitting (*β* = 0). We have made the electric dipole approximation and the rotating-wave approximation in ${\hat{H}}_{\text{cav}-\text{mol}}$ . Here, ${\hat{\mu }}_{m}$ is the electric dipole operator associated with the **m**th molecule and ${\hat{E}}_{k,\alpha }\left(r,z\right)$ is the electric field operator of the mode with polarization *α* and in-plane wave vector **k**. In addition, ***μ***_*α*′_ ⋅ **J**_**k**,*α*_ is the collective coupling strength of the cavity mode labeled by **k**, *α* and the $\left|G\right\rangle$ to $\left|\alpha_{\text{mol}}^{\prime }\right\rangle$ transition of the molecules (see Section S1 in Supporting Information). In the Hamiltonian, we only include cavity modes with **k** that lies within the first Brillouin zone determined by the porphyrin lattice −*π*/*a* < *k*_*x*_, *k*_*y*_ < *π*/*a*. We ignore cavity modes with larger wavevectors (Umklapp terms) as they are off-resonant and would have a negligible effect on the bands of our interest.

The photon modes of an empty cavity experience TE-TM splitting due to polarization-dependent reflection from the mirrors [41]. While the TE-TM splitting lifts the degeneracy between photon modes at |**k**| ≠ 0, photon modes of both polarizations remain degenerate at |**k**| = 0 due to rotational symmetry of the cavity mirrors about the *z*-axis. However, for Berry curvature and Chern invariant to be well defined, we need the photon/polariton bands to be separated in energy at all **k**; to achieve this, we include the perylene crystal. The anisotropy and emergent optical activity of the perylene crystal lifts the degeneracy between the photon modes at all **k** [25].

To compute the Berry curvature and Chern number, we focus on the first excitation manifold, which is spanned by states $\left|m,\pm_{\text{mol}}\right\rangle ={\hat{\sigma }}_{m,\pm }^{†}\left|vac\right\rangle$ and $\left|k,\pm_{\text{cav}}\right\rangle ={\hat{a}}_{k,\pm }^{†}\left|vac\right\rangle$ . Here, $\left|vac\right\rangle$ is the absolute ground state of the system where the photon modes are empty and all molecules are in their ground states. Rewriting the Hamiltonian with operators ${\hat{\sigma }}_{k,\alpha }$ , where ${\hat{\sigma }}_{m,\alpha }=\frac{1}{\sqrt{N_{x}N_{y}}}\sum_{k\in BZ}{\text{e}}^{\text{i}k\cdot r_{m}}{\hat{\sigma }}_{k,\alpha }$ and restricting ourselves to the first excitation manifold, we find $\hat{H}\left(k\right)=\left\langle k\right|\hat{H}\left|k\right\rangle$ to be

(4) $$\hat{H}\left(k\right)={\hat{H}}_{\text{mol}}\left(k\right)+{\hat{H}}_{\text{cav}}\left(k\right)+{\hat{H}}_{\text{cav}-\text{mol}}\left(k\right),$$

where,

(5) $$\begin{array}{ll} {\hat{H}}_{\text{mol}}\left(k\right) & =\hbar \omega_{\text{e}}\left|{+}_{\text{mol}}\right\rangle \left\langle {+}_{\text{mol}}\right|+\hbar \omega_{\text{e}}\left|{-}_{\text{mol}}\right\rangle \left\langle {-}_{\text{mol}}\right|,\\ {\hat{H}}_{\text{cav}}\left(k\right) & =\left(E_{0}+\frac{\hbar^{2}|k{|}^{2}}{2m^{*}}+\zeta |k|\cos\phi \right)\left|{+}_{\text{cav}}\right\rangle \left\langle {+}_{\text{cav}}\right|\\ & \;+\left(E_{0}+\frac{\hbar^{2}|k{|}^{2}}{2m^{*}}-\zeta |k|\cos\phi \right)\left|{-}_{\text{cav}}\right\rangle \left\langle {-}_{\text{cav}}\right|\\ & \;+\left(-\beta_{0}+\beta |k{|}^{2}{\text{e}}^{-\text{i}2\phi }\right)\left|{+}_{\text{cav}}\right\rangle \left\langle {-}_{\text{cav}}\right|\\ & \;+\left(-\beta_{0}+\beta |k{|}^{2}{\text{e}}^{\text{i}2\phi }\right)\left|{-}_{\text{cav}}\right\rangle \left\langle {+}_{\text{cav}}\right|,\\ {\hat{H}}_{\text{cav}-\text{mol}}\left(k\right) & =J_{k,+}\cdot \left(\mu_{+}\left|{+}_{\text{mol}}\right\rangle +\mu_{-}\left|{-}_{\text{mol}}\right\rangle \right)\left\langle {+}_{\text{cav}}\right|\\ & \;+J_{k,-}\cdot \left(\mu_{+}\left|{+}_{\text{mol}}\right\rangle +\mu_{-}\left|{-}_{\text{mol}}\right\rangle \right)\left\langle {-}_{\text{cav}}\right|+H.c. \end{array}$$

Here, **k** lies within the first Brillouin zone determined by the porphyrin lattice *k*_*x*_, *k*_*y*_ ∈ [−*π*/*a*, *π*/*a*]. As we are only interested in length scales much larger than *a*, we take the continuum limit *a* → 0 while keeping *μ*_0_/*a* a constant. Therefore, terms such as the collective light–matter coupling strength, **J**_**k**,*α*_ ⋅ ***μ***_*α*′_, remain constant in this limit (see Section S1 in Supporting Information). Moreover, upon taking the continuum limit, $\hat{H}\left(k\right)$ does not change; only the range of **k** becomes infinitely large, $k_{x},k_{y}\in R$ , that is, our system acquires complete translational invariance in the *x*–*y* plane. For such continuous systems, since $k_{x},k_{y}\in R$ is unbounded, we need to map (*k*_*x*_, *k*_*y*_) onto a sphere, which is a closed and bounded surface using stereographic projection before we compute Chern numbers [42] (see Section S2 in Supporting Information).

**Figure 3:**
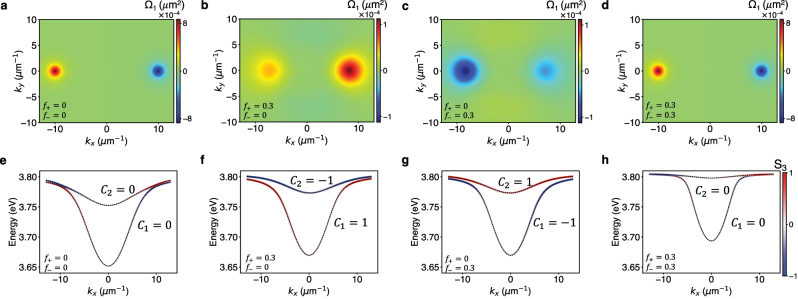
Berry curvature and degree of circular polarization of the bands. (a–d) Berry curvature of the lowest energy band, Ω_1_(**k**), and (e–h) a slice of the band structure at *k*_*y*_ = 0 of the lower two bands, under different levels of optical pumping, which create populations: (a, e) *f*_+_ = *f*_−_ = 0, (b, f) *f*_+_ = 0.3, *f*_−_ = 0, (c, g) *f*_+_ = 0, *f*_−_ = 0.3, and (d, h) *f*_+_ = *f*_−_ = 0.3. (e–h) The colors of the band indicate the value of the Stokes parameter, *S*_3_(**k**), which measures the degree of circular polarization of a mode (Eq. (8)). The Chern numbers *C*_1_ and *C*_2_ of the bands are also specified and are nonzero under time-reversal symmetry (TRS) breaking, that is, when *f*_+_ ≠ *f*_−_. We used parameters *β*_0_ = 0.1 eV, *β* = 9 × 10^−4^ eV μm^2^, *ζ* = 2.5 × 10^−3^ eV μm, m* = 125*ℏ* ^2^ eV^−1^ μm^−2^, *E*_0_ = 3.80 eV, and *ℏω*_e_ = 3.81 eV (see Section S4 in Supporting Information for details).

When we diagonalize the Hamiltonian in Eq. (5), we obtain four bands which we label with *l* = 1, 2, 3, 4 in increasing order of energy. In Figure 3a, we plot the Berry curvature, Ω_1_(**k**), of the lowest band *l* = 1, and in Figure 3e, we plot the *k*_*y*_ = 0 slice of the band structure of the two bands lowest in energy, *l* = 1, 2. As expected, in the absence of optical pumping, this system preserves TRS, which can be verified using the condition on Berry curvature Ω_*l*_(**k**) = −Ω_*l*_(−**k**), and the Chern numbers of the all the bands *C*_*l*_ = 0. Also, note that, the smallest splitting between the lower two bands within −13 μm^−1^ < *k*_*x*_, *k*_*y*_ < 13 μm^−1^ is ∼2.8 meV, which is larger than the linewidth of the transition in porphyrin at 4 K ( ∼0.5 meV) [43, 44].

### 2.2 Optical pumping

Optical pumping can saturate the electronic transitions of a system. This leads to reduction in the effective light–matter coupling strength, and, therefore, Rabi splitting contraction [11, 45, 46]. For instance, when the pump excites a fraction of molecules, *f*_E_, to the excited state and the remaining population stays in the ground state, *f*_G_, it results in Rabi splitting contraction proportional to $\sqrt{f_{\text{G}}-f_{\text{E}}}=\sqrt{1-2f_{\text{E}}}$ [47].

In our system, when the molecules are optically pumped, a fraction, *f*_+_, of the molecules occupy the $\left|{+}_{\text{mol}}\right\rangle$ state, another fraction, *f*_−_, occupy the $\left|{-}_{\text{mol}}\right\rangle$ state, and the remaining fraction, *f*_G_, are in the ground state $\left|G\right\rangle$ . The Rabi contraction corresponding to the $\left|G\right\rangle$ to $\left|{+}_{\text{mol}}\right\rangle$ transition should then be proportional to $\sqrt{f_{\text{G}}-f_{+}}$ which equals $\sqrt{1-f_{-}-2f_{+}}$ since *f*_G_ + *f*_+_ + *f*_−_ = 1. Similarly, the contraction should be proportional to $\sqrt{1-f_{+}-2f_{-}}$ for the $\left|G\right\rangle$ to $\left|{-}_{\text{mol}}\right\rangle$ transition. This difference in light–matter coupling when *f*_+_ ≠ *f*_−_ effectively introduces 2D chirality into the system [48].

To derive an effective Hamiltonian under optical pumping, we use Heisenberg equations of motion and make a mean-field approximation following the approach of Ribeiro et al. [47] (see Section S3 in Supporting Information). We then obtain the effective Hamiltonian,

(6) $${\hat{H}}^{\text{eff}}\left(k\right)={\hat{H}}_{\text{mol}}^{\text{eff}}\left(k\right)+{\hat{H}}_{\text{cav}}^{\text{eff}}\left(k\right)+{\hat{H}}_{\text{cav}-\text{mol}}^{\text{eff}}\left(k\right),$$

where,

(7) $$\begin{array}{ll} {\hat{H}}_{\text{mol}}^{\text{eff}}\left(k\right) & =\hbar \omega_{\text{e}}{\left|{+}_{\text{mol}}\right\rangle }^{\prime }{\left\langle {+}_{\text{mol}}\right|}^{\prime }+\hbar \omega_{\text{e}}{\left|{-}_{\text{mol}}\right\rangle }^{\prime }{\left\langle {-}_{\text{mol}}\right|}^{\prime },\\ {\hat{H}}_{\text{cav}}^{\text{eff}}\left(k\right) & =\left(E_{0}+\frac{\hbar^{2}|k{|}^{2}}{2m^{*}}+\zeta |k|\cos\phi \right){\left|{+}_{\text{cav}}\right\rangle }^{\prime }{\left\langle {+}_{\text{cav}}\right|}^{\prime }\\ & \;+\left(E_{0}+\frac{\hbar^{2}|k{|}^{2}}{2m^{*}}-\zeta |k|\cos\phi \right)\\ & \;\times {\left|{-}_{\text{cav}}\right\rangle }^{\prime }{\left\langle {-}_{\text{cav}}\right|}^{\prime }\\ & \;+\left(-\beta_{0}+\beta |k{|}^{2}{\text{e}}^{-\text{i}2\phi }\right){\left|{+}_{\text{cav}}\right\rangle }^{\prime }{\left\langle {-}_{\text{cav}}\right|}^{\prime }\\ & \;+\left(-\beta_{0}+\beta |k{|}^{2}{\text{e}}^{\text{i}2\phi }\right){\left|{-}_{\text{cav}}\right\rangle }^{\prime }{\left\langle {+}_{\text{cav}}\right|}^{\prime },\\ {\hat{H}}_{\text{cav}-\text{mol}}^{\text{eff}}\left(k\right) & =J_{k,+}\cdot \left(\sqrt{1-f_{-}-2f_{+}}\mu_{+}{\left|{+}_{\text{mol}}\right\rangle }^{\prime }\right.\\ & \;+\left.\sqrt{1-f_{+}-2f_{-}}\mu_{-}{\left|{-}_{\text{mol}}\right\rangle }^{\prime }\right){\left\langle {+}_{\text{cav}}\right|}^{\prime }\\ & \;+J_{k,-}\cdot \left(\sqrt{1-f_{-}-2f_{+}}\mu_{+}{\left|{+}_{\text{mol}}\right\rangle }^{\prime }\right.\\ & \;+\left.\sqrt{1-f_{+}-2f_{-}}\mu_{-}{\left|{-}_{\text{mol}}\right\rangle }^{\prime }\right)\\ & \;\times {\left\langle {-}_{\text{cav}}\right|}^{\prime }+H.c. \end{array}$$

Here, the states ${\left|\gamma \right\rangle }^{\prime }$ are different from states $\left|\gamma \right\rangle$ in Eq. (5), where *γ* = ±_mol_, ±_cav_. As expected, the light–matter coupling terms are scaled by factors $\sqrt{1-f_{\mp }-2f_{\pm }}$ , which is a consequence of the commutation relation in Eq. (1) (see Section S3 in Supporting Information).

If the pump pulse is circularly polarized, *f*_+_ ≠ *f*_−_, the Rabi contraction factor that multiplies the light–matter coupling differs for transitions to the $\left|{+}_{\text{mol}}\right\rangle$ and $\left|{-}_{\text{mol}}\right\rangle$ states; as a result, time-reversal symmetry is broken. Consequently, when *f*_+_ > *f*_−_, we find that bands 1 and 2 have nonzero Chern numbers +1 and −1 (Figure 3f). Under the opposite condition, *f*_+_ < *f*_−_, the Chern numbers reverse sign as seen in Figure 3g. When *f*_+_ = *f*_−_, TRS is preserved, and all bands have Chern number 0 as seen in Figure 3e and h. In Figure 3b and c, we plot the computed Berry curvature when *f*_+_ ≠ *f*_−_ and due to broken TRS, we find Ω_*l*_(**k**) ≠ −Ω_*l*_(−**k**). Nonzero values of Berry curvature are found at *k*_*x*_ ∼±8 μm^−1^, *k*_*y*_ ∼ 0 μm^−1^ when *f*_+_ = 0.3, *f*_−_ = 0 or *f*_+_ = 0, *f*_−_ = 0.3. To measure the Berry curvature of the bands at these values of **k**, the linewidths of the cavity modes and the molecular transitions need to be less than 10 meV as the energy splittings between the bands are 10 − 15 meV.

**Figure 4:**
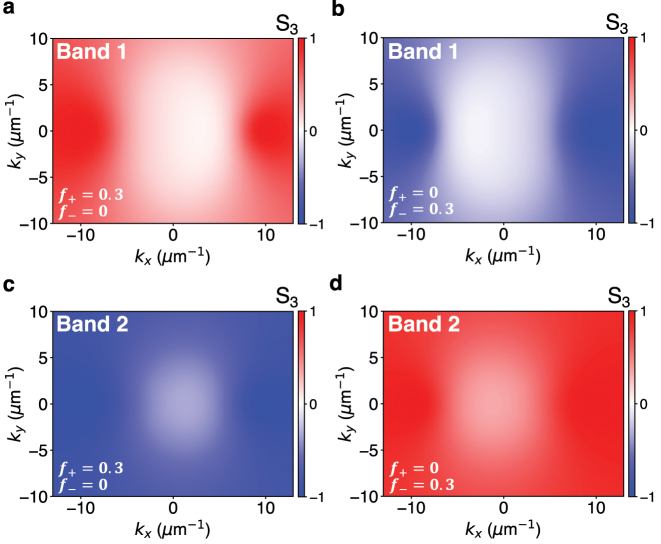
The Stokes parameter, *S*_3_(**k**), which is a measure of the degree of circular polarization of a mode (Eq. (8)), under pumping with (a, c) *σ*_+_ polarized light, which creates populations *f*_+_ = 0.3, *f*_−_ = 0, and (b, d) *σ*_−_ polarized light, which creates populations *f*_+_ = 0, *f*_−_ = 0.3 of the two lowest energy bands (band 1 and 2 as indicated in the inset). We used parameters *β*_0_ = 0.1 eV, *β* = 9 × 10^−4^ eV μm^2^, *ζ* = 2.5 × 10^−3^ eV μm, m* = 125*ℏ* ^2^ eV^−1^ μm^−2^, *E*_0_ = 3.80 eV, and *ℏω*_e_ = 3.81 eV (see Section S4 in Supporting Information for details).

We also plot the Stokes parameter, *S*_3_(**k**), for bands 1 and 2, under pumping with circularly polarized light, in Figure 4. The Stokes parameter, *S*_3_(**k**), provides information on the degree of circular polarization of the photonic component of an exciton–polariton band and is calculated as

(8) $$S_{3}\left(k\right)=\frac{|b_{+,cav}\left(k\right){|}^{2}-|b_{-,cav}\left(k\right){|}^{2}}{|b_{+,cav}\left(k\right){|}^{2}+|b_{-,cav}\left(k\right){|}^{2}}$$

where the eigenvectors of the band are $\left|u_{l,k}\right\rangle =b_{+,cav}\left(k\right)\left|{+}_{\text{cav}}\right\rangle +b_{-,cav}\left(k\right)\left|{-}_{\text{cav}}\right\rangle +b_{+,mol}\left(k\right)\left|{+}_{\text{mol}}\right\rangle +b_{-,mol}\left(k\right)\left|{-}_{\text{mol}}\right\rangle$ . In the absence of pumping, we find that within a band, one half of the modes are predominantly *σ*_+_ polarized and the other half are *σ*_−_ polarized (Figure 3e). Upon pumping with circularly polarized light, a large number of modes within each band gradually become of the same polarization as |*f*_+_ − *f*_−_| is increased (Figure 3f and g, Figures 4 and S2).

In experiments, the Berry curvature of photon bands in a Fabry–Perot cavity can be extracted from the components of the Stokes vector [25, 26]. In the case of exciton–polariton bands, the Berry curvature can be measured experimentally using the Stokes vector when the bands of the system can be separated into pairs of bands that are effectively described by separate 2 × 2 Hamiltonians. At each **k**, the Stokes vector can describe a state in a two-dimensional Hilbert space; however, the Stokes vector does not contain enough information to fully specify a state in a Hilbert space of dimensions larger than two. Therefore, in our four band model, the Berry curvature (Figure 3a–d) can be experimentally measured by pump-probe spectroscopy only when the splitting induced by the light–matter coupling is much larger than that induced by the coupling between cavity modes because then the four polariton bands can be separated into two pairs of bands that are effectively described by separate 2 × 2 Hamiltonians as in ref. [49]. This measurement should be feasible as long as the time delay between the pump and probe pulses is shorter than the time the system takes to depolarize and reach a state with *f*_+_ = *f*_−_. The system’s depolarization time depends only upon the bare molecular depolarization rate. As the depolarization timescale for porphyrins ranges from 210 fs to 1.6 ps, this measurement should be viable [50].

Population imbalances in the molecule or solid-state system can potentially be experimentally created in a variety of ways. One possibility is to directly excite higher energy material transitions with circularly polarized light that are within the transparency window of the cavity typically known as “nonresonant” pumping [11, 51]. If decay from those higher energy transitions into the relevant excited states happens before depolarization ensues, we will have obtained the desired population imbalances. Another possibility that bypasses the need of incoherent processes is a stimulated electronic Raman scattering with circularly polarized fields, although this scenario might require X-rays [52, 53]. Finally, the population imbalance may also be created by pumping resonantly with a circularly polarized laser at $|k|=\sqrt{\beta_{0}/\beta }$ , *ϕ* = 0. At this angle, the coupling between the circularly polarized cavity modes is zero. Additionally, |**J**_**k**,+_ ⋅ ***μ***_−_|≫|**J**_**k**,+_ ⋅ ***μ***_+_| and |**J**_**k**,−_ ⋅ ***μ***_+_|≫|**J**_**k**,−_ ⋅ ***μ***_−_| for all |**k**| ≪ *n*_*z*_ *π*/*L*_*z*_. Therefore, when the polariton mode at this **k** is pumped with circularly polarized light, the cavity mode of only the corresponding circular polarization is excited and population is transferred largely to only one of the circularly polarized molecular states. After dephasing into the molecular states (but not depolarization of the latter), the populations of the molecular states would be unequal *f*_+_ ≠ *f*_−_.

As the Chern numbers of bands 1 and 2 are modified through pumping with circularly polarized light, if we perform a calculation where a region of the system is pumped with *σ*_+_ polarized light (*f*_+_ ≠ 0 and *f*_−_ = 0) and an adjacent region is pumped with *σ*_−_ polarized light (*f*_+_ = 0 and *f*_−_ ≠ 0), we expect edge states at the boundary between these regions. However, as our Hamiltonian does not contain couplings between neighboring molecules, and the position of a molecule does not enter the Hamiltonian anywhere except through the phase of the light–matter coupling ${\text{e}}^{\text{i}{k\cdot r}_{m}}$ , the standard bulk-boundary correspondence is no longer applicable and we do not observe edge states. We do not include plots for these calculations in this work and leave it an open question whether there is an analogous statement for bulk-boundary correspondence in these types of systems. On the other hand, for exciton–polariton systems where nearest-neighbor couplings are present, edge states have been predicted and observed [19, 20].

### 2.3 Other systems

**Figure 5:**
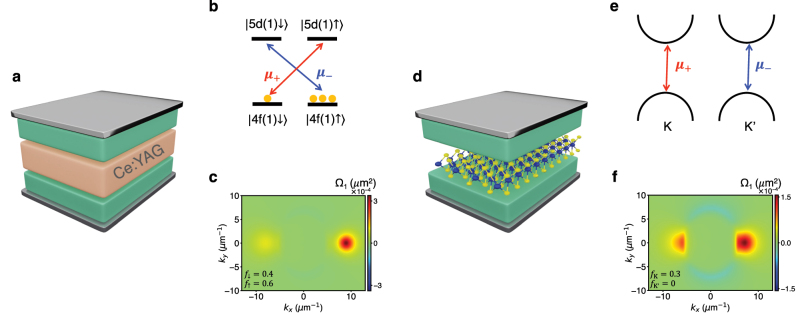
Solid-state polariton systems where population imbalance induces non-trivial topology. (a) Illustration of Ce:YAG (salmon block) and perylene (green blocks) within a Fabry–Perot cavity. (b) Atomic levels of Ce^3+^ ions embedded in yttrium aluminum garnet (YAG) where the yellow circles indicate the fraction *f*_↓_ of Ce^3+^ ions in the $\left|4f\left(1\right)↓\right\rangle$ state and the fraction *f*_↑_ in the $\left|4f\left(1\right)↑\right\rangle$ state after optical pumping. The transition dipoles $\mu_{\pm }=\mu_{0}\left(\hat{x}\pm i\hat{y}\right)/\sqrt{2}$ are also indicated. (c) Berry curvature of the lowest energy band, Ω_1_(**k**), under pumping with circularly polarized which creates populations *f*_↓_ = 0.4 and *f*_↑_ = 0.6. (d) Illustration of monolayer MoS_2_ and perylene (green blocks) within a Fabry–Perot cavity. (e) Illustration of A-excitons in the K and K′ valleys of monolayer MoS_2_. (f) Berry curvature of the lowest energy band, Ω_1_(**k**), under pumping with circularly polarized which creates exciton populations *f*_K_ = 0.3 and *f*_K′_ = 0. We used parameters *β*_0_ = 0.1 eV, *β* = 9 × 10^−4^ eV μm^2^, *ζ* = 2.5 × 10^−3^ eV μm, m* = 125*ℏ* ^2^ eV^−1^ μm^−2^, (c) *E*_0_ = 2.50 eV, *ℏω*_e_ = 2.53 eV and (f) *E*_0_ = 1.80 eV, *ℏω*_e_ = 1.855 eV (see Section S4 in Supporting Information for details).

To emphasize that our scheme of saturating electronic transitions with circularly polarized light to modify topological properties is not limited to organic exciton–polariton systems, we compute the Berry curvature of two other polariton systems where porphyrin is replaced with (i) Ce:YAG and (ii) MoS_2_ (Figure 5a and d). Other materials can also be used in place of porphyrins, as long as they have transitions that can be selectively excited with circularly polarized light and these transitions have large enough transition dipole moments that they can couple strongly to the photon modes of a cavity.

In yttrium aluminum garnet (YAG) doped with cerium, Ce^3+^ ions replace some Y^3+^ and Ce^3+^ has transitions that can be selectively excited with circularly polarized light. Here, each Ce^3+^ has two possible ground states, one with the electron in spin up $\left|4f\left(1\right)↑\right\rangle$ , and the other with it in spin down $\left|4f\left(1\right)↓\right\rangle$ . Similarly, it has a degenerate pair of excited spin states $\left|5d\left(1\right)↑\right\rangle$ and $\left|5d\left(1\right)↓\right\rangle$ . The $\left|4f\left(1\right)↓\right\rangle ↔\left|5d\left(1\right)↑\right\rangle$ transition has ∼400 times larger oscillator strength for excitation with *σ*_+_ polarized light than with *σ*_−_ polarized light; therefore, we take the transition dipole moment to be ***μ***_+_ (Figure 5b) [54]. Similarly, we take the transition dipole to be ***μ***_−_ for the $\left|4f\left(1\right)↑\right\rangle ↔\left|5d\left(1\right)↓\right\rangle$ transition (Figure 5b). The transitions in Ce:YAG do couple to photon modes, however, to the best of our knowledge, strong coupling has not been reported in the literature [55, 56]. Nevertheless, strong light–matter coupling has been achieved with a similar system: Nd^3+^ doped YSO and YVO crystals [57, 58], and based on our calculations, with a 0.1 μm thick sample of Ce:YAG at concentration 1 % Ce^3+^ (relative to Y^3+^), we should be able to attain strong coupling with photon modes in a Fabry–Perot cavity (see Section S4 in Supporting Information).

Under thermal equilibrium, the populations of the $\left|4f\left(1\right)↑\right\rangle$ and $\left|4f\left(1\right)↓\right\rangle$ states are equal. However, under pumping with pulses of *σ*_+_ polarization, in the presence of a small magnetic field ∼0.049 T, the population of $\left|4f\left(1\right)↑\right\rangle$ will exceed that of $\left|4f\left(1\right)↓\right\rangle$ because population is selectively removed from $\left|4f\left(1\right)↓\right\rangle$ and added to $\left|5d\left(1\right)↑\right\rangle$ by the circularly polarized pulses, but decay from the excited $\left|5d\left(1\right)↑\right\rangle$ state to the two ground states has equal probability [59]. In principle, a magnetic field is not required; however, as we do not know the spin relaxation time in the absence of the magnetic field, we report the magnetic field used in the experimental study [59]. Under optical pumping with circularly polarized light, the 5*d* states will have very small populations, which we take to be zero, while the $\left|4f\left(1\right)↓\right\rangle$ and $\left|4f\left(1\right)↑\right\rangle$ states will have unequal populations *f*_↓_ and *f*_↑_, respectively; here, *f*_↓_ + *f*_↑_ = 1. Optically pumped Ce:YAG can then be modeled using the effective Hamiltonian in Eqs. (6) and (7), with ${\left|\pm_{\text{mol}}\right\rangle }^{\prime }\to \left|5d\left(1\right)↑/↓\right\rangle$ and $\sqrt{1-f_{\mp }-2f_{\pm }}\to \sqrt{f_{↓/↑}}$ . The large spin relaxation time of ∼4.5 ms makes this system particularly well suited for our scheme because it maintains *f*_↓_ ≠ *f*_↑_, and hence nonzero Chern invariants, for an extended period of time [59]. In Figure 5c, we plot Berry curvature of the lowest band of a perylene filled cavity strongly coupled with Ce:YAG, where *f*_↓_ = 0.4 and *f*_↑_ = 0.6 (see Section S4 in Supporting Information for values of other parameters).

TMDs, such as single-layer MoS_2_, display optically controllable valley polarization and could also be used in place of porphyrins [60–62]. Due to lack of inversion symmetry in these systems, the K and K′ valleys are inequivalent; this results in optical selection rules that allow selective creation of excitons at K and K′ valleys with *σ*_+_ and *σ*_−_ polarized light, respectively [63, 64]. Additionally, strong light–matter coupling has been observed when monolayer MoS_2_ is placed within a Fabry–Perot cavity [8, 9]. This system has depolarization times of ∼200 fs to 5 ps making it possible to measure Berry curvature using pump-probe spectroscopy before depolarization occurs [65, 66]. We model this exciton–polariton system (Figure 5d) using Eqs. (6) and (7) (we focus on the A-exciton, see Section S4 in Supporting Information for parameters) with $\left|{+}_{\text{mol}}\right\rangle \to \left|K\right\rangle$ , $\left|{-}_{\text{mol}}\right\rangle \to \left|K^{\prime }\right\rangle$ and $\sqrt{1-f_{\mp }-2f_{\pm }}\to \sqrt{1-2f_{\text{K}/K^{\prime }}}$ . In Figure 5f, we plot the Berry curvature of the lowest band when *f*_K_ = 0.3 and *f*_K′_ = 0. Unfortunately, significant Rabi contraction upon optical pumping has not been experimentally observed in these systems, which will make it challenging to observe Berry curvature as in Figure 5f since our model relies on saturation effects. However, for exciton–polaritons formed from monolayer TMDs, even if Rabi contraction through resonant optical pumping may not produce the intended effect, off-resonant optical pumping can break the degeneracy of excitons in the K and K′ valleys through optical stark effect [67], and this may have interesting consequences for the Berry curvature. Additionally, if bilayer MoS_2_ is used in place of monolayer MoS_2_, effects on the Berry curvature described in our work may be more pronounced as bilayer MoS_2_ hosts interlayer excitons, which possess large optical nonlinearities; specifically, they display saturation and Rabi contraction under strong coupling [68, 69].

Finally, so far we have only considered replacing porphyrin with a different material, such as MoS_2_ or Ce:YAG. In addition to this, perylene can also be replaced with other suitable materials. In our work, we choose to use a cavity filled with perylene because we do not want degeneracy at any **k** within the photon bands. Other systems also satisfy this requirement and could be used instead. For instance, we could use an electrically tunable, highly anisotropic, liquid-crystal cavity with well-separated H and V polarized photon modes [24, 70]. A perovskite cavity is another potential candidate due to its high anisotropy, and optical pumping may help lift the degeneracy of polariton modes in this system [49]. Additionally, other photonic structures can also be used instead of a cavity, as long as the photon bands are not degenerate at any **k** and have nonzero light–matter coupling at all **k**.

In our analysis, we have disregarded the explicit role of vibrational modes, which is a reasonable assumption for rigid molecular systems (such as porphyrins and phthalocyanines [71]) and solid-state systems as their electron–phonon (vibronic) couplings tend to be small.

## 3 Conclusions

In summary, we show that TRS can be broken in organic exciton–polariton systems through selectively saturating electronic transitions with a circularly polarized pump and that the resulting bands possess nonzero Chern invariants. In particular, we demonstrate this theoretically for a Fabry–Perot cavity filled with porphyrin and perylene. The Berry curvature of the more photonic parts of the bands of this system can be measured experimentally using pump-probe spectroscopy, as long as the time delay is shorter than the depolarization time for porphyrin (210 fs to 1.6 ps) [50], and this will reveal nonzero Berry curvature and Chern number under circularly polarized pumping. Our scheme relies on Rabi contraction from saturation of optical transitions. It is important to note that edge states do not emerge in our system despite nonzero Chern invariants as our model does not contain sufficient positional information about the molecules or the unit cells. Bleu et al. [30] have previously proposed breaking TRS in inorganic exciton–polariton systems through pumping with circularly polarized light; however, their work relies on polariton condensation and having patterned lattices. Finally, we demonstrate that saturating electronic transitions to modify topology is not limited to organic systems. To illustrate this, we calculate the Berry curvature and Chern numbers of exciton–polariton bands of two other systems under optical pumping: (a) Ce:YAG and (b) monolayer MoS_2_, and find similar results as the organic exciton–polariton case. In view of recent developments on electrically tuning the Berry curvature of liquid-crystal and perovskite-filled cavities [24, 26], our work provides an additional control knob to optically tune the Berry curvature of exciton–polariton systems using circularly polarized light. Additionally, ultrafast control of topological properties of systems with light may find use in nonreciprocal and nonlinear optoelectronic devices.

## Supplementary Material

Supplementary Material Details
