# Supplementary material for: Molecular and solid-state topological polaritons induced by population imbalance
Source: Nanophotonics. 2023 Jun 12;12(15):3109–19. doi: 10.1515/nanoph-2023-0158 (PMC11501679; doi:10.1515/nanoph-2023-0158)
Supplement: Supplementary file 1 — Supplementary Material Details [file j_nanoph-2023-0158_suppl_001.pdf]

Supplementary Material: Molecular and solid-state topological polaritons induced by population imbalance

SINDHANA PANNIR-SIVAJOTHI[1], NATHANIEL P. STERN[2], AND JOEL YUEN-ZHOU[1,*]

[1] Department of Chemistry and Biochemistry, University of California San Diego , La Jolla, California 92093, USA
[2] Department of Physics and Astronomy, Northwestern University, Evanston, Illinois 60208, USA

S1. LIGHT-MATTER COUPLING

The light-matter coupling part of the total Hamiltonian under the electric dipole approximation is,

$$\begin{aligned}\hat{H}_{\text{cav-mol}} &= \sum_{\mathbf{m}}\sum_{\mathbf{k},\alpha} -\hat{\boldsymbol{\mu}}_{\mathbf{m}} \cdot \hat{\mathbf{E}}_{\mathbf{k},\alpha}(\mathbf{r}_{\mathbf{m}}, 0), \\ &= \sum_{\mathbf{m}}\sum_{\mathbf{k},\alpha} -\Big[\sum_{\alpha'=\pm}(\boldsymbol{\mu}_{\alpha'}\hat{\sigma}^{\dagger}_{\mathbf{m},\alpha'} + \boldsymbol{\mu}^{*}_{\alpha'}\hat{\sigma}_{\mathbf{m},\alpha'})\Big] \cdot \hat{\mathbf{E}}_{\mathbf{k},\alpha}(\mathbf{r}_{\mathbf{m}}, 0),\end{aligned} \tag{S1}$$

where $\boldsymbol{\mu}_{\alpha'} = \boldsymbol{\mu}_{\mathbf{m},\alpha'} = \langle \mathbf{m}, \alpha'_{\text{mol}} | \hat{\boldsymbol{\mu}} | \mathbf{m}, \text{G}\rangle$ is independent of $\mathbf{m}$ since we assume that all porphyrin molecules lie flat in the x-y plane and are oriented. The electric field operator of the mode labeled by $\mathbf{k}$ and α is

$$\hat{\mathbf{E}}_{\mathbf{k},\alpha}(\mathbf{r}, z) = \sqrt{\frac{\hbar\omega_{\mathbf{k},\alpha}}{2V\varepsilon\epsilon_0}}\Big(\mathbf{f}^{*}_{\mathbf{k},\alpha}(\mathbf{r}, z)\hat{a}^{\dagger}_{\mathbf{k},\alpha} + \mathbf{f}_{\mathbf{k},\alpha}(\mathbf{r}, z)\hat{a}_{\mathbf{k},\alpha}\Big). \tag{S2}$$

Here, $V = L_x L_y L_z$ is the volume of the box we consider, where as mentioned in the main manuscript, we apply periodic boundary conditions along the x and y directions. From here on, we will call the in-plane area of the box $A = L_x L_y$. Here, $\mathbf{f}_{\mathbf{k},\alpha}(\mathbf{r}, z)$ is the mode profile and it satisfies [1]

$$\int d\mathbf{r} \int_0^{L_z} dz \mathbf{f}^{*}_{\mathbf{k},\alpha}(\mathbf{r}, z)\mathbf{f}_{\mathbf{k},\alpha}(\mathbf{r}, z) = L_z A. \tag{S3}$$

For the TE and TM modes [2],

$$\begin{aligned}\mathbf{f}_{\mathbf{k},\text{TE}}(\mathbf{r}, z) &= e^{i\mathbf{k}\cdot\mathbf{r}}\sqrt{2}\sin\left[\frac{n_z\pi}{L_z}\left(z + \frac{L_z}{2}\right)\right]\hat{\boldsymbol{\phi}}, \\ \mathbf{f}_{\mathbf{k},\text{TM}}(\mathbf{r}, z) &= e^{i\mathbf{k}\cdot\mathbf{r}}\sqrt{\frac{2}{|\mathbf{k}|^2 + (\frac{n_z\pi}{L_z})^2}}\left\{\left(\frac{n_z\pi}{L_z}\right)\sin\left[\frac{n_z\pi}{L_z}\left(z + \frac{L_z}{2}\right)\right]\hat{\boldsymbol{\rho}} - i|\mathbf{k}|\cos\left[\frac{n_z\pi}{L_z}\left(z + \frac{L_z}{2}\right)\right]\hat{\mathbf{z}}\right\}.\end{aligned} \tag{S4}$$

We make the rotating-wave approximation,

$$\begin{aligned}\hat{H}_{\text{cav-mol}} &= \sum_{\mathbf{m}}\sum_{\mathbf{k},\alpha} -\Big[\sum_{\alpha'=\pm}(\boldsymbol{\mu}_{\alpha'}\hat{\sigma}^{\dagger}_{\mathbf{m},\alpha'} + \boldsymbol{\mu}^{*}_{\alpha'}\hat{\sigma}_{\mathbf{m},\alpha'})\Big] \cdot \Big[\sqrt{\frac{\hbar\omega_{\mathbf{k},\alpha}}{2V\varepsilon\epsilon_0}}\Big(\mathbf{f}^{*}_{\mathbf{k},\alpha}(\mathbf{r}_{\mathbf{m}}, 0)\hat{a}^{\dagger}_{\mathbf{k},\alpha} + \mathbf{f}_{\mathbf{k},\alpha}(\mathbf{r}_{\mathbf{m}}, 0)\hat{a}_{\mathbf{k},\alpha}\Big)\Big], \\ &\approx \sum_{\mathbf{m},\alpha'}\sum_{\mathbf{k},\alpha} -\sqrt{\frac{\hbar\omega_{\mathbf{k},\alpha}}{2V\varepsilon\epsilon_0}}\Big[\boldsymbol{\mu}_{\alpha'} \cdot \mathbf{f}_{\mathbf{k},\alpha}(\mathbf{r}_{\mathbf{m}}, 0)\hat{\sigma}^{\dagger}_{\mathbf{m},\alpha'}\hat{a}_{\mathbf{k},\alpha} + \boldsymbol{\mu}^{*}_{\alpha'} \cdot \mathbf{f}^{*}_{\mathbf{k},\alpha}(\mathbf{r}_{\mathbf{m}}, 0)\hat{\sigma}_{\mathbf{m},\alpha'}\hat{a}^{\dagger}_{\mathbf{k},\alpha}\Big], \\ &= \sum_{\mathbf{m},\alpha'}\sum_{\mathbf{k},\alpha}\Big[\frac{e^{i\mathbf{k}\cdot\mathbf{r}_{\mathbf{m}}}}{\sqrt{N_x N_y}}(\boldsymbol{\mu}_{\alpha'} \cdot \mathbf{J}_{\mathbf{k},\alpha})\hat{\sigma}^{\dagger}_{\mathbf{m},\alpha'}\hat{a}_{\mathbf{k},\alpha} + \frac{e^{-i\mathbf{k}\cdot\mathbf{r}_{\mathbf{m}}}}{\sqrt{N_x N_y}}(\boldsymbol{\mu}^{*}_{\alpha'} \cdot \mathbf{J}^{*}_{\mathbf{k},\alpha})\hat{\sigma}_{\mathbf{m},\alpha'}\hat{a}^{\dagger}_{\mathbf{k},\alpha}\Big],\end{aligned} \tag{S5}$$

where $\mathbf{J}_{\mathbf{k},\alpha} = -\sqrt{N_x N_y}\sqrt{\frac{\hbar\omega_{\mathbf{k},\alpha}}{2V\varepsilon\epsilon_0}}e^{-i\mathbf{k}\cdot\mathbf{r}}\mathbf{f}_{\mathbf{k},\alpha}(\mathbf{r}, 0)$ and $\boldsymbol{\mu}_{\alpha'} \cdot \mathbf{J}_{\mathbf{k},\alpha}$ is the collective light-matter coupling strength.

*e-mail: joelyuen@ucsd.edu

The annihilation operators of photon modes polarized along the horizontal (H) or x-axis and vertical (V) or y-axis are $\hat{a}_{\mathbf{k},\mathrm{H}}$ and $\hat{a}_{\mathbf{k},\mathrm{V}}$, respectively. They are related to $\alpha = \pm$ polarized modes through $\hat{a}_{\mathbf{k},\pm} = \frac{1}{\sqrt{2}}(\hat{a}_{\mathbf{k},\mathrm{H}} \mp i\hat{a}_{\mathbf{k},\mathrm{V}})$ [3]. In addition, we assume that they are related to the TM and TE modes through $\hat{a}_{\mathbf{k},\mathrm{TM}} = \cos\phi\hat{a}_{\mathbf{k},\mathrm{H}} + \sin\phi\hat{a}_{\mathbf{k},\mathrm{V}}$ and $\hat{a}_{\mathbf{k},\mathrm{TE}} = -\sin\phi\hat{a}_{\mathbf{k},\mathrm{H}} + \cos\phi\hat{a}_{\mathbf{k},\mathrm{V}}$. Using this, we obtain the relationship between $\hat{a}_{\mathbf{k},\mathrm{TE}}$, $\hat{a}_{\mathbf{k},\mathrm{TM}}$ and $\hat{a}_{\mathbf{k},+}$, $\hat{a}_{\mathbf{k},-}$ modes to be,

$$\begin{aligned}\hat{a}_{\mathbf{k},\mathrm{TM}} &= \frac{1}{\sqrt{2}}\Big(e^{i\phi}\hat{a}_{\mathbf{k},+} + e^{-i\phi}\hat{a}_{\mathbf{k},-}\Big),\\ \hat{a}_{\mathbf{k},\mathrm{TE}} &= \frac{1}{\sqrt{2}}\Big(ie^{i\phi}\hat{a}_{\mathbf{k},+} - ie^{-i\phi}\hat{a}_{\mathbf{k},-}\Big).\end{aligned} \tag{S6}$$

It is important to note that, based on these relationships and S4, the α =H/V modes are not completely linearly polarized and the $\alpha = \pm$ modes are not completely circularly polarized when $|\mathbf{k}|$ becomes comparable with $n_z\pi/L_z$. We also find,

$$\begin{aligned}\mathbf{J}_{\mathbf{k},+} &= \frac{e^{i\phi}}{\sqrt{2}}\Big(\mathbf{J}_{\mathbf{k},\mathrm{TM}} + i\mathbf{J}_{\mathbf{k},\mathrm{TE}}\Big),\\ \mathbf{J}_{\mathbf{k},-} &= \frac{e^{-i\phi}}{\sqrt{2}}\Big(\mathbf{J}_{\mathbf{k},\mathrm{TM}} - i\mathbf{J}_{\mathbf{k},\mathrm{TE}}\Big).\end{aligned} \tag{S7}$$

To keep the collective coupling strength $\boldsymbol{\mu}_{\alpha'} \cdot \mathbf{J}_{\mathbf{k},\alpha}$ constant while taking the $a \to 0$ limit, we take the magnitude of the collective transition dipole of the bright state $\sqrt{N_x N_y}\mu_0$ over square root of the quantization area of the photon mode $\sqrt{A}$ to be a constant; that is, we keep $\sqrt{\rho_A}\mu_0 = \mu_0/a$ a constant, where $\rho_A = N_x N_y/A$ is the areal density of quantum emitters.

$$\begin{aligned}\mathbf{J}_{\mathbf{k},\alpha} &= -\sqrt{\rho_A}\sqrt{\frac{\hbar\omega_{\mathbf{k},\alpha}}{2L_z\varepsilon\epsilon_0}}e^{-i\mathbf{k}.\mathbf{r}}\mathbf{f}_{\mathbf{k},\alpha}(\mathbf{r},0)\\ &= -\frac{1}{a}\sqrt{\frac{\hbar\omega_{\mathbf{k},\alpha}}{2L_z\varepsilon\epsilon_0}}e^{-i\mathbf{k}.\mathbf{r}}\mathbf{f}_{\mathbf{k},\alpha}(\mathbf{r},0).\end{aligned} \tag{S8}$$

S2. CHERN NUMBER CALCULATION

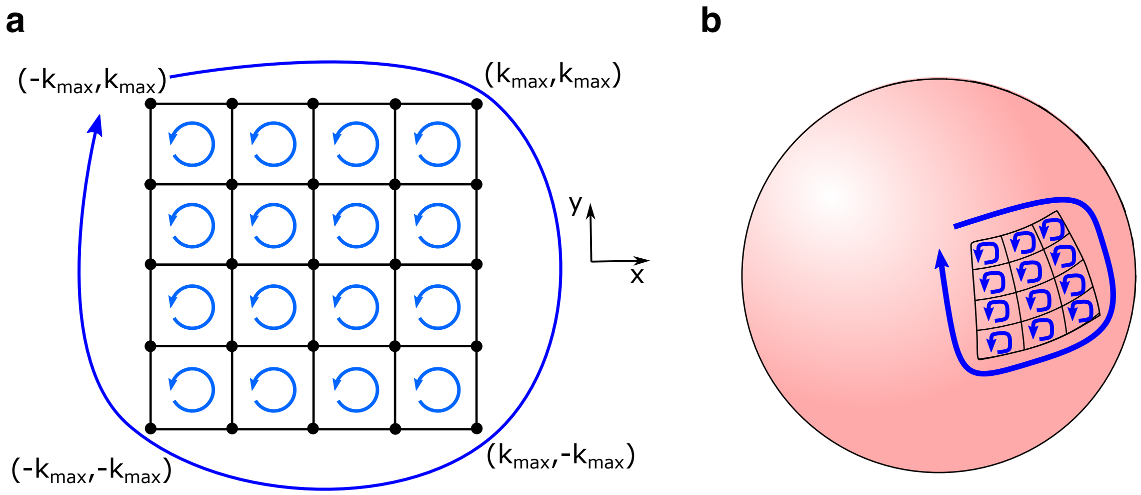

Fig. S1. (a) This is a cartoon figure that demonstrates the way Berry flux and Chern number are computed in our system. The small squares are the plaquettes over which Berry flux is computed. The blue arrows specify the orientation used for Berry flux computation. Note that the direction is opposite for the small squares and the large square. (b) Same as (a), but placed on a sphere. Here, it is more clear that the direction of the arrow for the large square indicates the way Berry flux is computed for the giant plaquette covering the rest of the sphere.

For the Chern invariant to be an integer, it is important that the Berry curvature is integrated over a closed and bounded surface [4]. For periodic systems with a finite period, the Brillouin zone is a torus which satisfies this requirement. However, for a continuous system, (k_x, k_y) lies on an unbounded plane; for such systems, Silveirinha

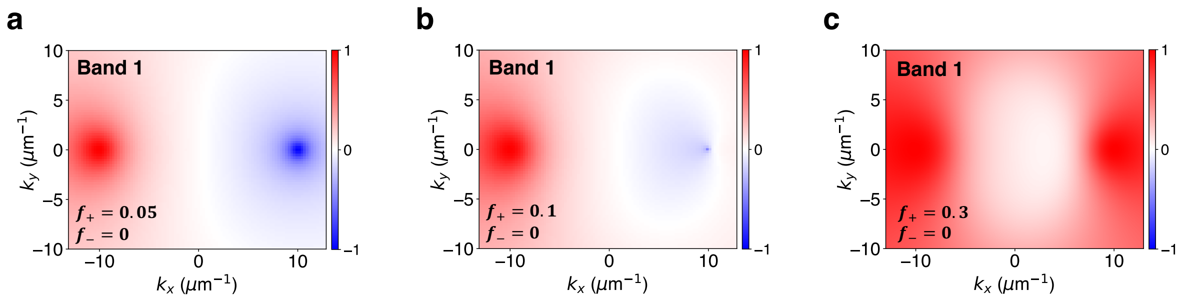

Fig. S2. The Stokes parameter, $S_3(\mathbf{k})$, of the lowest energy band (Band 1) under pumping with σ_+ polarized light which creates populations (a) $f_+ = 0.05$, $f_- = 0$, (b) $f_+ = 0.1$, $f_- = 0$, and (c) $f_+ = 0.3$, $f_- = 0$.

[5] proposed mapping this infinitely large plane onto a sphere to compute the Chern number. This is the procedure we follow in our work. We discretize k-space and compute the Berry flux in each plaquette within a square-shaped region in k-space, $-k_{\max} \le k_x, k_y \le k_{\max}$ [4, 6] (Fig. S1a and S1b). The entire region that satisfies the condition $k_x, k_y > k_{\max}$ or $k_x, k_y < -k_{\max}$ is taken as a single giant plaquette (Fig. S1b), and the Berry flux within this region is computed by taking the Berry phase along the boundary of the plaquette but in a direction opposite to that used to compute Berry flux for plaquettes within the square $-k_{\max} \le k_x, k_y \le k_{\max}$ as indicated in Fig. S1a and S1b. To ensure that we obtain a converged Chern number, we calculate the Chern number for different $k_{\max}$ and find that, for our system, once $k_{\max} \gtrsim 100\mu\text{m}^{-1}$, the Chern number converges to $C_1 = \pm 1, C_2 = \mp 1, C_3 = 0$, and $C_4 = 0$ when $f_+ \neq f_-$ with $|f_+ - f_-| \gtrsim 0.11$. Smaller differences between f_+ and f_-, $|f_+ - f_-| \lesssim 0.11$ require larger $k_{\max}$ for convergence. This is not a problem for the $f_+ = f_-$ case because the Chern invariant will always be zero due to time-reversal symmetry $\Omega_l(\mathbf{k}) = -\Omega_l(-\mathbf{k})$, and we can use $k_{\max} \approx 100\mu\text{m}^{-1}$ to compute it.

S3. OPTICAL PUMPING

The number of excitations in the system $N_{\text{ex}} = \sum_{\mathbf{k},\alpha} a^\dagger_{\mathbf{k},\alpha} a_{\mathbf{k},\alpha} + \sum_{\mathbf{n},\alpha} \sigma^\dagger_{\mathbf{n},\alpha} \sigma_{\mathbf{n},\alpha}$ is a conserved quantity of this Hamiltonian. Therefore, when we have f_+ fraction of molecules in the $|+_{\text{mol}}\rangle$ state and f_- in the $|-_{\text{mol}}\rangle$ state, we will only have to look at the $(f_+ + f_-)N^{\text{th}}$ excitation manifold. Unfortunately, the dimensions of the Hilbert space of this manifold scale as $\binom{N}{(f_+ + f_-)N}$, and this quickly becomes computationally intractable as the system size, N, increases. Using mean-field theory, we reduce this many-body problem to a one-body problem. That is, we derive an effective Hamiltonian for a single excitation in the mean-field of the remaining $(f_+ + f_-)N$ excitations; in this way, we reduce the dimensions of the Hilbert space to that of the first excitation manifold. To do this, we follow a procedure similar to that used by Ribeiro *et al.* [7] and write the Heisenberg equations of motion (EOM) for the operators $\hat{\sigma}_{\mathbf{m},\pm}$ and $\hat{a}_{\mathbf{k},\pm}$,

$$
\begin{aligned}
i\hbar \frac{d\hat{\sigma}_{\mathbf{n},\pm}}{dt} =& \big[\hat{\sigma}_{\mathbf{n},\pm}, \hat{H}_{\text{mol}}\big] + \big[\hat{\sigma}_{\mathbf{n},\pm}, \hat{H}_{\text{cav}}\big] + \big[\hat{\sigma}_{\mathbf{n},\pm}, \hat{H}_{\text{cav-mol}}\big] \\
=& \hbar\omega_{\text{e}}\hat{\sigma}_{\mathbf{n},\pm} + \frac{1}{\sqrt{N_x N_y}} \sum_{\mathbf{k}} e^{i\mathbf{k}\cdot\mathbf{r_n}} \Big[\big(1 - \hat{\sigma}^\dagger_{\mathbf{n},\mp}\hat{\sigma}_{\mathbf{n},\mp} - 2\hat{\sigma}^\dagger_{\mathbf{n},\pm}\hat{\sigma}_{\mathbf{n},\pm}\big)\Big(\mathbf{J}_{\mathbf{k},+}\cdot\boldsymbol{\mu}_\pm \hat{a}_{\mathbf{k},+} \\
&+ \mathbf{J}_{\mathbf{k},-}\cdot\boldsymbol{\mu}_\pm \hat{a}_{\mathbf{k},-}\Big) - \hat{\sigma}^\dagger_{\mathbf{n},\mp}\hat{\sigma}_{\mathbf{n},\pm}\Big(\mathbf{J}_{\mathbf{k},+}\cdot\boldsymbol{\mu}_\mp \hat{a}_{\mathbf{k},+} + \mathbf{J}_{\mathbf{k},-}\cdot\boldsymbol{\mu}_\mp \hat{a}_{\mathbf{k},-}\Big)\Big], \\
i\hbar \frac{d\hat{a}_{\mathbf{k},\pm}}{dt} =& \big[\hat{a}_{\mathbf{k},\pm}, \hat{H}_{\text{mol}}\big] + \big[\hat{a}_{\mathbf{k},\pm}, \hat{H}_{\text{cav}}\big] + \big[\hat{a}_{\mathbf{k},\pm}, \hat{H}_{\text{cav-mol}}\big] \\
=& \Big(E_0 + \frac{\hbar^2|\mathbf{k}|^2}{2m^*} \pm \zeta|\mathbf{k}|\cos\phi\Big)\hat{a}_{\mathbf{k},\pm} + \Big(-\beta_0 + \beta|\mathbf{k}|^2 e^{\mp i2\phi}\Big)\hat{a}_{\mp,\mathbf{k}} \\
&+ \frac{1}{\sqrt{N_x N_y}} \sum_{\mathbf{m}} e^{i\mathbf{k}\cdot\mathbf{r_m}} \Big(\mathbf{J}^*_{\mathbf{k},\pm}\cdot\boldsymbol{\mu}^*_+ \hat{\sigma}_{\mathbf{m},+} + \mathbf{J}^*_{\mathbf{k},\pm}\cdot\boldsymbol{\mu}^*_- \hat{\sigma}_{\mathbf{m},-}\Big).
\end{aligned}
\tag{S9}
$$

We make a mean-field approximation to linearize these EOM. For instance, we use $mn \approx \bar{m}n$, that is,

$$
\begin{aligned}
\hat{\sigma}^\dagger_{\mathbf{n},+}\hat{\sigma}_{\mathbf{n},+}\hat{a}_{\mathbf{k},+} =& \Big(\langle\hat{\sigma}^\dagger_{\mathbf{n},+}\hat{\sigma}_{\mathbf{n},+}\rangle + \hat{\sigma}^\dagger_{\mathbf{n},+}\hat{\sigma}_{\mathbf{n},+} - \langle\hat{\sigma}^\dagger_{\mathbf{n},+}\hat{\sigma}_{\mathbf{n},+}\rangle\Big)\hat{a}_{\mathbf{k},+} \\
=& \langle\hat{\sigma}^\dagger_{\mathbf{n},+}\hat{\sigma}_{\mathbf{n},+}\rangle\hat{a}_{\mathbf{k},+} + \big(\hat{\sigma}^\dagger_{\mathbf{n},+}\hat{\sigma}_{\mathbf{n},+} - \langle\hat{\sigma}^\dagger_{\mathbf{n},+}\hat{\sigma}_{\mathbf{n},+}\rangle\big)\langle\hat{a}_{\mathbf{k},+}\rangle \\
\approx& \langle\hat{\sigma}^\dagger_{\mathbf{n},+}\hat{\sigma}_{\mathbf{n},+}\rangle\hat{a}_{\mathbf{k},+},
\end{aligned}
\tag{S10}
$$

where $\langle \hat{O} \rangle = \mathrm{Tr}\left[\hat{\rho}_0 \hat{O}\right]$ with $\hat{\rho}_0 \approx \prod_{\mathbf{m}} \hat{\rho}_{\mathbf{m}} \prod_{\mathbf{k}} \prod_{\alpha=+,-} \hat{\rho}_{\alpha,\mathbf{k}}$ [8]. Here, we assume that after dephasing of the molecular amplitudes, $\hat{\rho}_{\mathbf{m}} = f_G \left|\mathbf{m}, G\right\rangle \left\langle \mathbf{m}, G\right| + f_+ \left|\mathbf{m}, +_{\mathrm{mol}}\right\rangle \left\langle \mathbf{m}, +_{\mathrm{mol}}\right| + f_- \left|\mathbf{m}, -_{\mathrm{mol}}\right\rangle \left\langle \mathbf{m}, -_{\mathrm{mol}}\right|$, $\hat{\rho}_{\alpha,\mathbf{k}} = \left|\mathbf{k}, \alpha_{\mathrm{cav}}, 0\right\rangle \left\langle \mathbf{k}, \alpha_{\mathrm{cav}}, 0\right|$, and, therefore, $\langle \hat{a}_{\mathbf{k},+} \rangle = 0$. The EOM then become

$$\begin{aligned}
i\hbar \frac{d\hat{\sigma}_{\mathbf{n},\pm}}{dt} \approx& \hbar\omega_{\mathrm{e}} \hat{\sigma}_{\mathbf{n},\pm} + \frac{1}{\sqrt{N_x N_y}} (1 - f_\mp - 2f_\pm) \sum_{\mathbf{k}} e^{i\mathbf{k}\cdot\mathbf{r}_{\mathbf{n}}} \Big(\mathbf{J}_{\mathbf{k},+} \cdot \boldsymbol{\mu}_\pm \hat{a}_{\mathbf{k},+} \\
&+ \mathbf{J}_{\mathbf{k},-} \cdot \boldsymbol{\mu}_\pm \hat{a}_{\mathbf{k},-} \Big), \\
i\hbar \frac{d\hat{a}_{\mathbf{k},\pm}}{dt} =& \Big(E_0 + \frac{\hbar^2 |\mathbf{k}|^2}{2m^*} \pm \zeta |\mathbf{k}| \cos\phi \Big) \hat{a}_{\mathbf{k},\pm} + \Big(-\beta_0 + \beta |\mathbf{k}|^2 e^{\mp i2\phi} \Big) \hat{a}_{\mp,\mathbf{k}} \\
&+ \frac{1}{\sqrt{N_x N_y}} \sum_{\mathbf{m}} e^{i\mathbf{k}\cdot\mathbf{r}_{\mathbf{m}}} \Big(\mathbf{J}^*_{\mathbf{k},\pm} \cdot \boldsymbol{\mu}^*_+ \hat{\sigma}_{\mathbf{m},+} + \mathbf{J}^*_{\mathbf{k},\pm} \cdot \boldsymbol{\mu}^*_- \hat{\sigma}_{\mathbf{m},-} \Big).
\end{aligned} \tag{S11}$$

We define rescaled operators $\hat{\sigma}'_{\mathbf{n},\pm} = \hat{\sigma}_{\mathbf{n},\pm} / \sqrt{1 - f_\mp - 2f_\pm}$ and rewrite the EOM,

$$\begin{aligned}
i\hbar \frac{d\hat{\sigma}'_{\mathbf{n},\pm}}{dt} \approx& \hbar\omega_{\mathrm{e}} \hat{\sigma}'_{\mathbf{n},\pm} + \frac{1}{\sqrt{N_x N_y}} \sqrt{1 - f_\mp - 2f_\pm} \sum_{\mathbf{k}} e^{i\mathbf{k}\cdot\mathbf{r}_{\mathbf{n}}} \Big(\mathbf{J}_{\mathbf{k},+} \cdot \boldsymbol{\mu}_\pm \hat{a}_{\mathbf{k},+} \\
&+ \mathbf{J}_{\mathbf{k},-} \cdot \boldsymbol{\mu}_\pm \hat{a}_{\mathbf{k},-} \Big), \\
i\hbar \frac{d\hat{a}_{\mathbf{k},\pm}}{dt} =& \Big(E_0 + \frac{\hbar^2 |\mathbf{k}|^2}{2m^*} \pm \zeta |\mathbf{k}| \cos\phi \Big) \hat{a}_{\mathbf{k},\pm} + \Big(-\beta_0 + \beta |\mathbf{k}|^2 e^{\mp i2\phi} \Big) \hat{a}_{\mp,\mathbf{k}} \\
&+ \frac{}{\sqrt{N_x N_y}} \sum_{\mathbf{m}} e^{i\mathbf{k}\cdot\mathbf{r}_{\mathbf{m}}} \Big(\sqrt{1 - f_- - 2f_+} \mathbf{J}^*_{\mathbf{k},\pm} \cdot \boldsymbol{\mu}^*_+ \hat{\sigma}'_{\mathbf{m},+} + \sqrt{1 - f_+ - 2f_-} \mathbf{J}^*_{\mathbf{k},\pm} \cdot \boldsymbol{\mu}^*_- \hat{\sigma}'_{\mathbf{m},-} \Big).
\end{aligned} \tag{S12}$$

From these EOM, along with the fact that $\hat{\sigma}'_{\mathbf{n},\pm}$ act effectively as bosonic operators in mean-field, $\left[\hat{\sigma}'_{\mathbf{n},+}, \hat{\sigma}'^{\dagger}_{\mathbf{n},+}\right] = \frac{1 - \hat{\sigma}^{\dagger}_{\mathbf{n},-}\hat{\sigma}_{\mathbf{n},-} - 2\hat{\sigma}^{\dagger}_{\mathbf{n},+}\hat{\sigma}_{\mathbf{n},+}}{1 - f_- - 2f_+} \approx \hat{I}$ and $\left[\hat{\sigma}'_{\mathbf{n},+}, \hat{\sigma}'^{\dagger}_{\mathbf{n},-}\right] = \frac{-\hat{\sigma}^{\dagger}_{\mathbf{n},-}\hat{\sigma}_{\mathbf{n},+}}{1 - f_- - 2f_+} \approx \hat{0}$, where $\hat{I}$ and $\hat{0}$ are the identity and zero operators, we can construct an effective Hamiltonian $\hat{H}^{\mathrm{eff}} = \hat{H}^{\mathrm{eff}}_{\mathrm{mol}} + \hat{H}^{\mathrm{eff}}_{\mathrm{cav}} + \hat{H}^{\mathrm{eff}}_{\mathrm{cav-mol}}$ in $\hat{\sigma}'_{\mathbf{n},\pm}$ and $\hat{a}_{\mathbf{k},\pm}$,

$$\begin{aligned}
\hat{H}^{\mathrm{eff}}_{\mathrm{mol}} =& \sum_{\mathbf{n}} \Big(\hbar\omega_{\mathrm{e}} \hat{\sigma}'^{\dagger}_{\mathbf{n},+} \hat{\sigma}'_{\mathbf{n},+} + \hbar\omega_{\mathrm{e}} \hat{\sigma}'^{\dagger}_{\mathbf{n},-} \hat{\sigma}'_{\mathbf{n},-} \Big), \\
\hat{H}^{\mathrm{eff}}_{\mathrm{cav}} =& \sum_{\mathbf{k}} \Big(E_0 + \frac{\hbar^2 |\mathbf{k}|^2}{2m^*} + \zeta |\mathbf{k}| \cos\phi \Big) \hat{a}^{\dagger}_{\mathbf{k},+} \hat{a}_{\mathbf{k},+} \\
&+ \Big(E_0 + \frac{\hbar^2 |\mathbf{k}|^2}{2m^*} - \zeta |\mathbf{k}| \cos\phi \Big) \hat{a}^{\dagger}_{\mathbf{k},-} \hat{a}_{\mathbf{k},-} + \Big(-\beta_0 + \beta |\mathbf{k}|^2 e^{-i2\phi} \Big) \hat{a}^{\dagger}_{\mathbf{k},+} \hat{a}_{\mathbf{k},-} \\
&+ \Big(-\beta_0 + \beta |\mathbf{k}|^2 e^{i2\phi} \Big) \hat{a}^{\dagger}_{\mathbf{k},-} \hat{a}_{\mathbf{k},+}, \\
\hat{H}^{\mathrm{eff}}_{\mathrm{cav-mol}} =& \frac{1}{\sqrt{N_x N_y}} \sum_{\mathbf{m}} \sum_{\mathbf{k}} e^{i\mathbf{k}\cdot\mathbf{r}_{\mathbf{m}}} \Bigg[\sqrt{1 - f_- - 2f_+} \Big(\mathbf{J}_{\mathbf{k},+} \cdot \boldsymbol{\mu}_+ \hat{\sigma}'^{\dagger}_{\mathbf{m},+} \hat{a}_{\mathbf{k},+} \\
&+ \mathbf{J}_{\mathbf{k},-} \cdot \boldsymbol{\mu}_+ \hat{\sigma}'^{\dagger}_{\mathbf{m},+} \hat{a}_{\mathbf{k},-} \Big) + \sqrt{1 - f_+ - 2f_-} \Big(\mathbf{J}_{\mathbf{k},+} \cdot \boldsymbol{\mu}_- \hat{\sigma}'^{\dagger}_{\mathbf{m},-} \hat{a}_{\mathbf{k},+} \\
&+ \mathbf{J}_{\mathbf{k},-} \cdot \boldsymbol{\mu}_- \hat{\sigma}'^{\dagger}_{\mathbf{m},-} \hat{a}_{\mathbf{k},-} \Big) \Bigg] + \mathrm{H.c.},
\end{aligned} \tag{S13}$$

which is the mean-field Hamiltonian when the system has f_+, f_- excitations. Writing this effective Hamiltonian in

k-space,

$$\begin{aligned}
\hat{H}^{\text{eff}}_{\text{mol}} =& \sum_{\mathbf{k}} \Big[\hbar\omega_{\text{e}}\hat{\sigma}'^{\dagger}_{\mathbf{k},+}\hat{\sigma}'_{\mathbf{k},+} + \hbar\omega_{\text{e}}\hat{\sigma}'^{\dagger}_{\mathbf{k},-}\hat{\sigma}'_{\mathbf{k},-}\Big],\\
\hat{H}^{\text{eff}}_{\text{cav}} =& \sum_{\mathbf{k}} \Big(E_0 + \frac{\hbar^2|\mathbf{k}|^2}{2m^*} + \zeta|\mathbf{k}|\cos\phi\Big)\hat{a}^{\dagger}_{\mathbf{k},+}\hat{a}_{\mathbf{k},+} + \Big(E_0 + \frac{\hbar^2|\mathbf{k}|^2}{2m^*} - \zeta|\mathbf{k}|\cos\phi\Big)\hat{a}^{\dagger}_{\mathbf{k},-}\hat{a}_{\mathbf{k},-}\\
&+ \Big(-\beta_0 + \beta|\mathbf{k}|^2 e^{-i2\phi}\Big)\hat{a}^{\dagger}_{\mathbf{k},+}\hat{a}_{\mathbf{k},-} + \Big(-\beta_0 + \beta|\mathbf{k}|^2 e^{i2\phi}\Big)\hat{a}^{\dagger}_{\mathbf{k},-}\hat{a}_{\mathbf{k},+},\\
\hat{H}^{\text{eff}}_{\text{cav-mol}} =& \sum_{\mathbf{k}} \Bigg[\sqrt{1-f_- - 2f_+}\Big(\mathbf{J}_{\mathbf{k},+}\cdot\boldsymbol{\mu}_+\hat{\sigma}'^{\dagger}_{\mathbf{k},+}\hat{a}_{\mathbf{k},+}\\
&+ \mathbf{J}_{\mathbf{k},-}\cdot\boldsymbol{\mu}_+\hat{\sigma}'^{\dagger}_{\mathbf{k},+}\hat{a}_{\mathbf{k},-}\Big) + \sqrt{1-f_+ - 2f_-}\Big(\mathbf{J}_{\mathbf{k},+}\cdot\boldsymbol{\mu}_-\hat{\sigma}'^{\dagger}_{\mathbf{k},-}\hat{a}_{\mathbf{k},+}\\
&+ \mathbf{J}_{\mathbf{k},-}\cdot\boldsymbol{\mu}_-\hat{\sigma}'^{\dagger}_{\mathbf{k},-}\hat{a}_{\mathbf{k},-}\Big)\Bigg] + \text{H.c.}
\end{aligned} \tag{S14}$$

We define states $|\mathbf{k}, \pm_{\text{mol}}\rangle'$ and $|\mathbf{k}, \pm_{\text{cav}}\rangle'$ corresponding to operators $\hat{\sigma}'^{\dagger}_{\mathbf{k},\pm}$ and $\hat{a}^{\dagger}_{\mathbf{k},\pm}$, respectively. Writing the Hamiltonian $\hat{H}^{\text{eff}}(\mathbf{k}) = \langle\mathbf{k}|\,\hat{H}^{\text{eff}}\,|\mathbf{k}\rangle$ in the above basis we obtain,

$$\hat{H}^{\text{eff}}(\mathbf{k}) = \hat{H}^{\text{eff}}_{\text{mol}}(\mathbf{k}) + \hat{H}^{\text{eff}}_{\text{cav}}(\mathbf{k}) + \hat{H}^{\text{eff}}_{\text{cav-mol}}(\mathbf{k}), \tag{S15}$$

where,

$$\begin{aligned}
\hat{H}^{\text{eff}}_{\text{mol}}(\mathbf{k}) =& \hbar\omega_{\text{e}}\,|+_{\text{mol}}\rangle'\,\langle+_{\text{mol}}|' + \hbar\omega_{\text{e}}\,|-_{\text{mol}}\rangle'\,\langle-_{\text{mol}}|',\\
\hat{H}^{\text{eff}}_{\text{cav}}(\mathbf{k}) =& \Big(E_0 + \frac{\hbar^2|\mathbf{k}|^2}{2m^*} + \zeta|\mathbf{k}|\cos\phi\Big)\,|+_{\text{cav}}\rangle'\,\langle+_{\text{cav}}|' + \Big(E_0 + \frac{\hbar^2|\mathbf{k}|^2}{2m^*} - \zeta|\mathbf{k}|\cos\phi\Big)\,|-_{\text{cav}}\rangle'\,\langle-_{\text{cav}}|'\\
&+ \Big(-\beta_0 + \beta|\mathbf{k}|^2 e^{-i2\phi}\Big)\,|+_{\text{cav}}\rangle'\,\langle-_{\text{cav}}|' + \Big(-\beta_0 + \beta|\mathbf{k}|^2 e^{i2\phi}\Big)\,|-_{\text{cav}}\rangle'\,\langle+_{\text{cav}}|',\\
\hat{H}^{\text{eff}}_{\text{cav-mol}}(\mathbf{k}) =& \mathbf{J}_{\mathbf{k},+}\cdot\Big(\sqrt{1-f_- - 2f_+}\boldsymbol{\mu}_+\,|+_{\text{mol}}\rangle' + \sqrt{1-f_+ - 2f_-}\boldsymbol{\mu}_-\,|-_{\text{mol}}\rangle'\Big)\,\langle+_{\text{cav}}|'\\
&+ \mathbf{J}_{\mathbf{k},-}\cdot\Big(\sqrt{1-f_- - 2f_+}\boldsymbol{\mu}_+\,|+_{\text{mol}}\rangle' + \sqrt{1-f_+ - 2f_-}\boldsymbol{\mu}_-\,|-_{\text{mol}}\rangle'\Big)\,\langle-_{\text{cav}}|' + \text{H.c.}
\end{aligned} \tag{S16}$$

Upon pumping with circularly polarized light, the lowest band gradually changes from containing equal number of modes of both circular polarizations to overwhelmingly containing modes of a single polarization as $|f_+ - f_-|$ increases (Fig. S2).

S4. PARAMETERS

Perylene filled cavity

We take parameters for the perylene filled cavity $\beta_0 = 0.1$eV, $\beta = 9\times10^{-4}$eVμm^2, $\zeta = 2.5\times10^{-3}$eVμm, $m^* = 125\hbar^2$eV$^{-1}\mu$m^{-2}, and $L_z = 0.745\mu$m, where these are similar to those used to model the experiments of Ren *et al.* [9] (Fig. 3, 4, and 5 in main manuscript). On the other hand, we modify E_0 and n_z such that they make the photon modes in our model near resonant with the transition that is strongly coupled to the cavity. For instance, we take $E_0 = 3.80$eV and $n_z = 11$ for porphyrin (Fig. 3 and 4); $E_0 = 2.50$eV and $n_z = 9$ for Ce:YAG (Fig. 5b-c); and $E_0 = 1.80$eV and $n_z = 5$ for MoS_2 (Fig. 5e-f). We assume that perylene has a similar effect on these different photon modes, as it does on modes with $E_0 \sim 2.27$eV at $\mathbf{k} = 0$ in experiments [9]. This may not necessarily be true, however, as we consider a perylene filled cavity only to achieve frequency separation of photon modes with different polarization, and this can instead be easily achieved with an electrically tunable liquid crystal cavity [10], replacing a perylene filled cavity with a liquid-crystal cavity will not modify the underlying physics of the phenomenon we are interested in, *i.e.*, the idea of using saturation to break TRS will remain intact.

Porphyrin, Ce:YAG, and monolayer MoS_2

We take areal density $\rho_A = 3.55\times10^5\mu$m^{-2} ($\sim$ 2000 molecules in 75nm $\times$ 75nm) [11], relative permittivity $\varepsilon = 1.5$ [12], frequency $\hbar\omega_{\text{e}} = 3.8056$eV and transition dipole $\mu_0 = 1.1184$au $\times$ 2.5417D/au $= 2.84$D [13] for the porphyrin film. Also, we consider 100 such porphyrin films stacked one over the other along the z direction within the cavity to achieve strong light-matter coupling, $N_z = 100$. Therefore, the effective areal density of molecules $\rho'_A = N_z\rho_A$ will be used instead of ρ_A while computing $\mathbf{J}_{\mathbf{k},\alpha}$. These are the parameters used to generate Fig. 3 and 4.

Similarly, using density $\rho_{\text{YAG}} = 5.11$g cm^{-3}, molar mass $M_{\text{YAG}} = 738$ g mol^{-1}, number of Y^{3+} per unit cell $n_{Y^{3+}} = 3$, and concentration of Ce^{3+} (relative to Y^{3+}) 1% $= 10^{-2}$ [14], we obtain the effective areal density of Ce^{3+}

ions in a $L'_z = 0.1\mu$m thick layer of Ce:YAG to be $\rho'_A = 10^{-2} L'_z n_{Y^{3+}} \rho_{YAG} N_A / M_{YAG} = 1.25 \times 10^7 \mu m^{-2}$. This will be used while computing $\mathbf{J}_{\mathbf{k},\alpha}$ in place of ρ_A. We use relative permittivity $\varepsilon = 12$ [15] and frequency $\hbar\omega_e = 2.53$eV (489nm [16]) for the transition in a Ce:YAG crystal. Using the oscillator strength of this transition 0.286 [16], we calculate the transition dipole $\mu_0 = 5.46$D. These are the parameters used to generate Fig. 5c.

For monolayer MoS_2, we consider A-excitons at $\hbar\omega_e = 1.855$eV [17]. From Chen *et al.* [17], we take the Rabi splitting at resonance, and use $\mu_0 \sqrt{\rho_A} \sqrt{\hbar\omega_e / 2L_z \varepsilon\epsilon_0} \approx 39\text{meV}/2 = 19.5$meV in our calculations (Fig. 5f).

REFERENCES

1. C. Fabre and N. Treps, "Modes and states in quantum optics," Rev. Mod. Phys. **92**, 035005 (2020).
2. H. Zoubi and G. C. La Rocca, "Microscopic theory of anisotropic organic cavity exciton polaritons," Phys. Rev. B **71**, 235316 (2005).
3. M. Martinelli and P. Martelli, "Polarization, mirrors, and reciprocity: birefringence and its compensation in optical retracing circuits," Adv. Opt. Photonics **9**, 129–168 (2017).
4. J. K. Asbóth, L. Oroszlány, and A. Pályi, *A short course on topological insulators* (Springer Cham, 2016).
5. M. G. Silveirinha, "Chern invariants for continuous media," Phys. Rev. B **92**, 125153 (2015).
6. T. Fukui, Y. Hatsugai, and H. Suzuki, "Chern numbers in discretized brillouin zone: Efficient method of computing (spin) hall conductances," J. Phys. Soc. Jpn. **74**, 1674–1677 (2005).
7. R. F. Ribeiro, A. D. Dunkelberger, B. Xiang, W. Xiong, B. S. Simpkins, J. C. Owrutsky, and J. Yuen-Zhou, "Theory for nonlinear spectroscopy of vibrational polaritons," J. Phys. Chem. Lett. **9**, 3766–3771 (2018).
8. P. Fowler-Wright, B. W. Lovett, and J. Keeling, "Efficient many-body non-markovian dynamics of organic polaritons," Phys. Rev. Lett. **129**, 173001 (2022).
9. J. Ren, Q. Liao, F. Li, Y. Li, O. Bleu, G. Malpuech, J. Yao, H. Fu, and D. Solnyshkov, "Nontrivial band geometry in an optically active system," Nat. Commun. **12**, 1–8 (2021).
10. K. Rechcińska, M. Król, R. Mazur, P. Morawiak, R. Mirek, K. Łempicka, W. Bardyszewski, M. Matuszewski, P. Kula, W. Piecek *et al.*, "Engineering spin-orbit synthetic Hamiltonians in liquid-crystal optical cavities," Science **366**, 727–730 (2019).
11. B. Hulsken, R. Van Hameren, J. W. Gerritsen, T. Khoury, P. Thordarson, M. J. Crossley, A. E. Rowan, R. J. Nolte, J. A. Elemans, and S. Speller, "Real-time single-molecule imaging of oxidation catalysis at a liquid–solid interface," Nat. Nanotechnol. **2**, 285–289 (2007).
12. D. Li, B. I. Swanson, J. M. Robinson, and M. A. Hoffbauer, "Porphyrin based self-assembled monolayer thin films: synthesis and characterization," J. Am. Chem. Soc. **115**, 6975–6980 (1993).
13. S. Sun, B. Gu, and S. Mukamel, "Polariton ring currents and circular dichroism of Mg-porphyrin in a chiral cavity," Chem. Sci. **13**, 1037–1048 (2022).
14. V. Bachmann, C. Ronda, and A. Meijerink, "Temperature quenching of yellow Ce^{3+} luminescence in YAG:Ce," Chem. Mater. **21**, 2077–2084 (2009).
15. P. Ctibor, J. Sedláček, and T. Hudec, "Dielectric properties of Ce-doped YAG coatings produced by two techniques of plasma spraying," Bol. Soc. Esp. Cerám. Vidr. **61**, 408–416 (2022).
16. R. Kolesov, K. Xia, R. Reuter, M. Jamali, R. Stöhr, T. Inal, P. Siyushev, and J. Wrachtrup, "Mapping spin coherence of a single rare-earth ion in a crystal onto a single photon polarization state," Phys. Rev. Lett. **111**, 120502 (2013).
17. Y.-J. Chen, J. D. Cain, T. K. Stanev, V. P. Dravid, and N. P. Stern, "Valley-polarized exciton–polaritons in a monolayer semiconductor," Nat. Photonics **11**, 431–435 (2017).
